# Parity modulates impact of BMI and gestational weight gain on gut microbiota in human pregnancy

**DOI:** 10.1080/19490976.2023.2259316

**Published:** 2023-10-09

**Authors:** Katherine M. Kennedy, Andreas Plagemann, Julia Sommer, Marie Hofmann, Wolfgang Henrich, Jon F.R. Barrett, Michael G. Surette, Stephanie Atkinson, Thorsten Braun, Deborah M. Sloboda

**Affiliations:** a Department of Biochemistry and Biomedical Sciences, McMaster University, Hamilton, Canada; b Department of Obstetrics and Gynecology, McMaster University, Hamilton, Canada; c Farncombe Family Digestive Health Research Institute, McMaster University, Hamilton, Canada; d Department of Obstetrics and Department of ‘Experimental Obstetrics’, Charité – Universitätsmedizin Berlin, corporate member of Freie Universität Berlin and Humboldt-Universität zu Berlin, Berlin, Germany; e Department of Medicine, McMaster University, Hamilton, Canada; f Department of Pediatrics, McMaster University, Hamilton, Canada

**Keywords:** microbiome, microbiota, microbial metabolites, parity, pregnancy, perinatal, metabolism

## Abstract

Dysregulation of maternal adaptations to pregnancy due to high pre-pregnancy BMI (pBMI) or excess gestational weight gain (GWG) is associated with worsened health outcomes for mothers and children. Whether the gut microbiome contributes to these adaptations is unclear. We longitudinally investigated the impact of pBMI and GWG on the pregnant gut microbiome. We show that the gut microbiota of participants with higher pBMI changed less over the course of pregnancy in primiparous but not multiparous participants. This suggests that previous pregnancies may have persistent impacts on maternal adaptations to pregnancy. This ecological memory appears to be passed on to the next generation, as parity modulated the impact of maternal GWG on the infant gut microbiome. This work supports a role of the gut microbiome in maternal adaptations to pregnancy and highlights the need for longitudinal sampling and accounting for parity as key considerations for studies of the microbiome in pregnancy and infants. Understanding how these factors contribute to and shape maternal health is essential for the development of interventions impacting the microbiome, including pre/probiotics.

## Background

Pregnancy requires appropriate metabolic adaptations to support fetal growth and development,^1^ including hyperglycemia, hyperinsulinemia, and increased adiposity. Dysregulation of these adaptations can occur in cases of high pre-pregnancy body mass index (pBMI) and/or excess gestational weight gain (GWG), leading to an increased risk of pregnancy complications including gestational diabetes and fetal macrosomia, and long-term metabolic dysfunction in both the mother and her child.^2^ Although some data suggest that pregnancy-associated metabolic adaptations may be influenced by shifts in the gut microbiota over the course of pregnancy,^3^ the few studies with longitudinal sampling present conflicting evidence. One of the first studies of the gut microbiome during pregnancy reported an increase in diversity between samples (beta diversity) and a decrease in diversity within samples (alpha diversity) in healthy pregnant individuals during the third trimester of pregnancy compared to the first.^4^ In contrast, others have found the maternal gut microbiota to be stable over the course of pregnancy.^5–9^ This absence of consistent evidence may be due to the large impact of *inter*individual variation.^10^ A longitudinal, *intra*individual evaluation of whether gut microbiota change temporally across pregnancy, birth, and the postnatal period is required to robustly assess whether host-microbe interactions could govern pregnancy adaptations.

There is growing evidence to suggest that the maternal gut microbiome plays an important role in the health of not only the mother, but also that of their child over the long term.^11^ Impaired maternal metabolic adaptations to pregnancy create an adverse *in utero* environment which alters fetal development; fetal alterations in the face of in utero adversity leads to significant changes in infant physiological responses to the postnatal environment. This ultimately leads to increased disease risk in adult offspring.^12^ For example, the infant gut, which is first colonized by maternal microbes during and after birth,^13,14^ relies on appropriate *in utero* intestinal development to achieve a postnatal intestinal niche that can select for commensal microbes beneficial for infant metabolic and immune health. Under adverse *in utero* circumstances, and/or neonatal exposure to a dysbiotic maternal microbiome, early-life gut development and gut microbiome establishment may be compromised. Therefore, it has been hypothesized that shifts in the maternal gut microbiome could play a role in the relationship between high pBMI/excess GWG and worsened health outcomes for both mother and child.

Although excess GWG is more likely to occur in cases where maternal pBMI is high, 50% of all pregnant individuals experience excess GWG.^15^ Excessive GWG can also contribute to postpartum weight retention, increasing the risk of high pBMI in subsequent pregnancies.^16^ Thus, these two factors both independently and together influence maternal and offspring health. So far, few studies have investigated the impact of high pBMI or excess GWG on the pregnant gut microbiota,^17–22^ and none have investigated the interaction of these impacts with pregnancy-associated microbial shifts longitudinally over the course of pregnancy and postpartum. Although prior pregnancies (parity) influence maternal metabolic adaptations to pregnancy^23^ to date, no human studies have investigated whether parity influences pregnancy-associated microbiotal shifts. In this prospective, observational cohort study, we set out to determine whether temporal changes in maternal gut microbial composition occur with advancing gestation and the postpartum period. We also investigated whether maternal BMI/GWG and parity impact maternal microbiotal adaptations and how these contexts influence maternal and infant microbiomes.

## Results

### Study cohort

A total of 89 participants were recruited; after we excluded participants who reported any nicotine or antibiotic use during pregnancy, 65 participants were included in the study. Of the included participants, 74% were German citizens and all participants were recruited at an urban clinic in Berlin, Germany. For clarity, we refer to participants for whom the study pregnancy was their first pregnancy reaching 24 weeks gestation as *primiparous* (nulliparous at enrollment and primiparous at study completion: *n* = 40). *Multiparous* participants (*n* = 25) had either one (88%) or two (12%) previous pregnancies reaching 24 weeks gestation. The majority of participants with a pre-pregnancy BMI (pBMI) of 25–30 or > 30 had gestational weight gain (GWG) above the Institute of Medicine (IOM) recommended range^16^ (excess GWG; 83% and 86% respectively), compared to less than half of participants with a pBMI 18.5–25 (33%; Table 1). A total of seven participants were diagnosed with gestational diabetes mellitus (GDM) during the study period, 2 of whom were treated with insulin. Most participants delivered vaginally (79%) and mode of delivery was similar across pBMI categories (q = 0.8) and did not differ by parity (q > 0.9; Table 1). Multiparous participants were older than primiparous participants (32.8 ± 3.1 vs 29.6 ± 4.4, q = 0.009), but there was no significant effect of parity or maternal age on pBMI, GWG, postpartum weight retention, length of gestation, or delivery mode. We analyzed dietary data collected in the first, second, and third trimester using a semiquantitative 25-item food frequency questionnaire (FFQ) adapted from the validated PrimeScreen FFQ.^24^ There was no significant effect of pBMI (Supplemental Table S1), GWG (Supplemental Table S2), or parity (Supplemental Table S3) on overall diet quality score or average weekly intake of any one FFQ item in any trimester after correction for multiple comparisons (FDR correction).

**Table 1.** Participant characteristics.

	Parity				Overall pBMI					Primiparous pBMI					Mutiparous pBMI				
Characteristic	Primiparous^1^	Multiparous^1^	p^2^	q^3^	<25^1^	25–30^1^	>30^1^	p^2^	q^1^	<25^1^	25–30^1^	>30^1^	p^2^	q^3^	<25^1^	25–30^1^	>30^1^	p^2^	q^3^
n	40	25			39	12	14			24	7	9			15	5	5		
Age	30 (4)	33 (3)	.002	0.009	31 (4)	31 (5)	30 (4)	.8	0.8	30 (5)	30 (5)	29 (3)	>.9	>0.9	33 (3)	34 (2)	32 (4)	.8	>0.9
Primigravida	27 (68%)	0 (0%)	<.001	<0.001	17 (44%)	3 (25%)	7 (50%)	.4	0.8	17 (71%)	3 (43%)	7 (78%)	.4	0.6	0 (0%)	0 (0%)	0 (0%)		
pBMI	23.8 (21.0, 29.0)	23.5 (21.2, 26.6)	>.9	>0.9	21.3 (19.6, 23.2)	26.4 (25.8, 28.4)	33.2 (31.3, 34.9)	<.001	<0.001	21.2 (19.4, 22.0)	28.0 (25.7, 28.8)	33.4 (32.7, 33.6)	<.001	<0.001	21.3 (20.2, 23.4)	26.1 (25.9, 26.6)	31.0 (30.9, 35.3)	<.001	<0.001
GWG (kg)	15.3 (6.1)	15.7 (5.3)	.8	>0.9	15.3 (4.7)	16.3 (7.4)	15.1 (7.0)	.6	0.8	14.9 (4.5)	16.8 (8.2)	15.1 (8.2)	.4	0.6	16.0 (5.1)	15.6 (7.1)	15.1 (5.0)	>.9	>0.9
GWG category			>.9	>.9				<0.001	.004				0.039	.2				0.066	.2
below	6 (15%)	3 (13%)			7 (19%)	2 (17%)	0 (0%)			5 (22%)	1 (14%)	0 (0%)			2 (15%)	1 (20%)	0 (0%)		
within	12 (31%)	6 (26%)			16 (44%)	0 (0%)	2 (14%)			10 (43%)	0 (0%)	2 (22%)			6 (46%)	0 (0%)	0 (0%)		
above	21 (54%)	14 (61%)			13 (36%)	10 (83%)	12 (86%)			8 (35%)	6 (86%)	7 (78%)			5 (38%)	4 (80%)	5 (100%)		
pp Weight Retained	3.1 (−.2, 7.6)	3.2 (2.0, 8.8)	.3	0.6	1.8 (0.3, 5.0)	4.7 (1.4, 8.1)	7.7 (3.1, 11.9)	.053	0.14	1.6 (−1.4, 3.8)	4.4 (1.0, 6.5)	7.8 (3.1, 12.4)	.083	0.2	2.5 (1.3, 7.4)	8.0 (2.7, 8.7)	6.0 (3.2, 9.9)	.5	>0.9
Length of gestation (days)	282 (274, 284)	277 (273, 282)	.3	0.6	280 (273, 284)	276 (272, 284)	279 (275, 288)	.7	0.8	282 (276, 284)	273 (270, 283)	282 (277, 289)	.5	0.6	277 (270, 282)	276 (275, 284)	278 (274, 279)	.9	>0.9
Delivery mode = vaginal	32 (80%)	18 (78%)	.9	>0.9	30 (81%)	10 (83%)	10 (71%)	.7	0.8	20 (83%)	5 (71%)	7 (78%)	.8	0.9	10 (77%)	5 (100%)	3 (60%)	.3	0.8

^1^n; Mean (SD); n (%); Median (IQR). ^2^Kruskal-Wallis rank sum test. ^1^False discovery rate correction for multiple testing.

### Parity modulates the impact of pBMI and GWG on maternal gut microbiota

Maternal stool samples were collected longitudinally: at the end of the first trimester (8–18 weeks gestation), second trimester (25–29 weeks gestation), and third trimester (29–38 weeks gestation) of pregnancy, at delivery, and at 6 months postpartum. We assessed the maternal gut microbiota using 16S ribosomal RNA gene sequencing of the combined V3-V4 region. We identified minimal sporadic contamination in negative controls, likely originating from samples. Of 13 sequenced gDNA extraction negative controls, 4 had no reads and an additional 4 had fewer than 20 reads. The 5 remaining negative controls had 45, 55, 80, 130, and 384 reads: 65 of 67 ASVs were detected in a single negative control (*Escherichia/Shigella* and *Bacteroides* were each detected in 2 negative controls). These negative control data show no consistent contamination signal. We identified 7577 ASVs in maternal samples after removal of non-bacterial reads (Kingdom Eukaryota, Order Chloroplast, Family Mitochondria, or no assigned Phylum) and the median sample read count was 62,853 (min = 14,872; max = 136,000).

**Figure 1.**
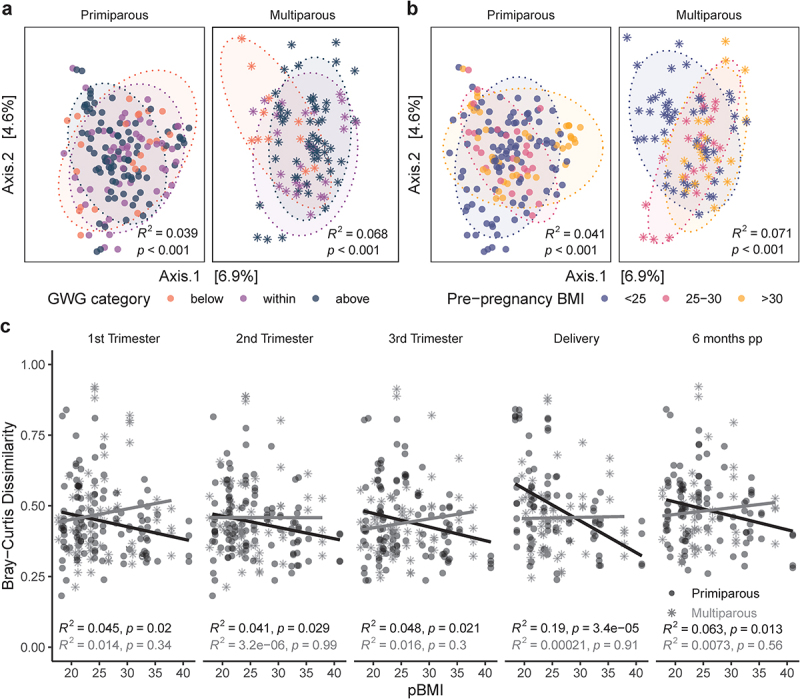
Parity modulates impact of pBMI and GWG on pregnant gut microbiota. PCoA of Bray-Curtis distances in primiparous (*n* = 39, one excluded – no GWG data) and multiparous (*n* = 23, two excluded – no GWG data) participants shows modest clustering by a, GWG category (primiparous, R2 = 0.039, *p* < .0001; multiparous R2 = .068, *p* < .0001) and b, pBMI category (primiparous, R2 = 0.041, *p* < .0001; multiparous R2 = 0.071, *p* < .0001). Significance was assessed by PERMANOVA blocked by sample time point as strata to account for repeated measures. c, Bray-Curtis dissimilarity at each timepoint to all other timepoints within individual participants are negatively associated with pBMI in primiparous participants (black trendline; *n* = 40), but not in multiparous participants (gray trendline; *n* = 25). Significance was analyzed within primiparous and multiparous participants by mixed linear model with pBMI, GWG category, and sample time point as interacting fixed effects and participant ID as a random effect.

Alpha diversity was positively correlated with pBMI (Shannon Index; R^2^ = 0.11, *p* = .010), particularly in participants with excess GWG overall (R^2^ = 0.12, *p* = .0069) and at each pregnancy time point (R^2^ = 0.14–0.21, *p* = 0.012–0.046; Supplemental Figure S1), but not at 6 months postpartum (R^2^ = 0.014, *p* = .57). To visualize differences in overall community composition between samples (beta diversity) we performed principal coordinate analysis (PCoA) of Bray-Curtis dissimilarity (Figure 1A,B). Beta diversity was modestly impacted by GWG category (PERMANOVA; R^2^ = 2.6%, *p* = .0001; Figure 1A), pBMI category (R^2^ = 2.8%, *p* = .0001; Figure 1B), parity (R^2^ = 1.4%, *p* = .0001; Supplemental Figure S2A), and sample time point (R^2^ = 1.0%, *p* = 0.0001; Supplemental Figure S2B). These modest effects were overshadowed by the impact of interindividual variability, which explained the majority of variation between samples (PERMANOVA; R^2^ = 62.3%, *p* = .0001). Longitudinal sampling within individuals is a powerful strategy for disentangling the large effects of *inter*individual variability,^25^ from factors that influence shifts in the microbiota associated with pregnancy (*intra*individual variability). We assessed intra-individual beta diversity (Bray-Curtis dissimilarity) longitudinally across all time points as a measure of how different the microbiota is at different time points within the same individual. We found that the impact of pBMI on beta diversity differed depending on parity. In primiparous participants, beta diversity was negatively correlated with pBMI for all time point comparisons (R^2^ = 0.041–0.19, *p* < 0.0001–0.029). In contrast, we found no significant correlation between beta diversity and pBMI in multiparous participants (R^2^ <0.0001–0.016, *p* = 0.3–.99; Figure 1C). These data suggest that in primiparous (first) pregnancy, a higher pBMI was associated with greater stability in the gut microbiota over the course of pregnancy and postpartum. Together these observations illustrate that when considering temporal changes in microbial composition related to pregnancy, longitudinal sampling within individuals is essential in uncovering patterns of change that exist with advancing gestation and persist postpartum. These data also highlight a novel observation; that gut microbial diversity during pregnancy and postpartum is influenced by previous pregnancies. Therefore, parity is a key confounder that must be considered in all future studies investigating the maternal microbiome.

### Parity modulates impact of pBMI and GWG on fecal SCFA levels

**Figure 2.**
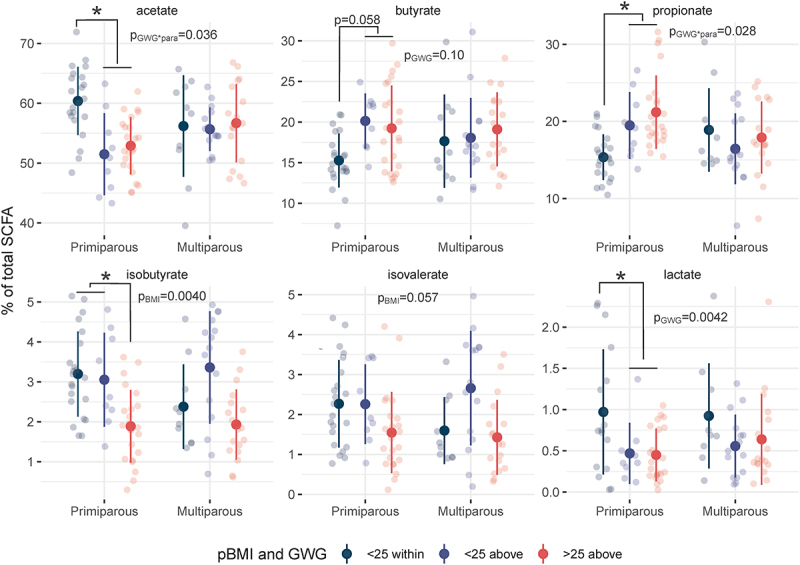
Relative SCFA concentrations differ with pBMI > 25 and excess GWG. dot plots of relative SCFA concentrations across all time points as a percentage of total SCFAs show a significant interaction of parity and GWG on fecal acetate (*p* = .036) and propionate (*p* = .028) driven by increased acetate and decreased propionate in primiparous participants with excess GWG. Excess GWG was also associated with decreased lactate (*p* = .0042), particularly in primiparous participants. A pBMI > 25 was associated with decreased isobutyrate (*p* = .0040). Primiparous (<25 within, *n* = 5; <25 above, *n* = 2; >25 above, *n* = 5); multiparous (<25 within, *n* = 3; <25 above, *n* = 3; >25 above, *n* = 4). Individual points are shown (transparent dots) as well as the mean (solid black dot) and standard deviation (whiskers). Significance was assessed by mixed linear model with pBMI category or GWG category as fixed effects and participant ID as a random effect.

Because of their functional role in metabolic signaling pathways^26^ we assessed fecal SCFA level associations with pBMI and GWG longitudinally across all time points in a subset of 97 maternal fecal samples (primiparous, *n* = 12; multiparous, *n* = 10; Figure 2). This subset included samples that had enough starting material for SCFA quantification and participants were selected for a balanced distribution of pBMI and GWG values. This subset was not biased in terms of parity, maternal age, gestational length, or mode of delivery compared to participants not included in SCFA analyses (Supplemental Table S4). To account for variability in recovery between samples, the relative concentration of each SCFA was assessed as a percentage of total SCFAs. Due to limited sample size, we assessed fecal SCFA levels by both GWG and pBMI – and the interaction of each of these factors with parity – across all samples.

Fecal SCFA levels differed by GWG, and this effect was dependent upon parity as differences occurred only in primiparous participants (Supplemental Tables S5 and S6). Fecal acetate was decreased by excess GWG (main effect of GWG-by-parity interaction *p* = .036) in primiparous participants (*p* = .0041), and this decrease was accompanied by an increase in propionate (*p* = .027, main effect of interaction *p* = .028). Overall, lactate was decreased in participants with excess GWG (*p* = .0042), particularly in primiparous participants (*p* = .0047). Together, these data suggest that differences in non-digestible carbohydrate fermentation processes that produce SCFAs, and by inference the biochemical pathways between them, appear to be driven by gestational weight gain in primiparous women.

In contrast, differences in branched SCFA (BCFA) levels were driven by pBMI. Overall, isobutyrate was decreased by pBMI > 25 (main effect of pBMI *p* = .0040 vs pBMI < 25), particularly in primiparous participants (*p* = .013). Previous studies have shown that BCFAs contribute to intestinal barrier function^27^ and facilitate the storage of diet-derived lipids in adipocytes by inhibiting lipolysis and increasing glucose uptake.^28^ Thus, decreased BCFAs may contribute to a decrease in appropriate maternal metabolic adaptive flexibility leading to increased risk of pregnancy complications in participants with a pBMI > 25 and excess GWG, although this hypothesis remains to be tested.

### Maternal taxonomic associations with pBMI, GWG, and parity

**Figure 3.**
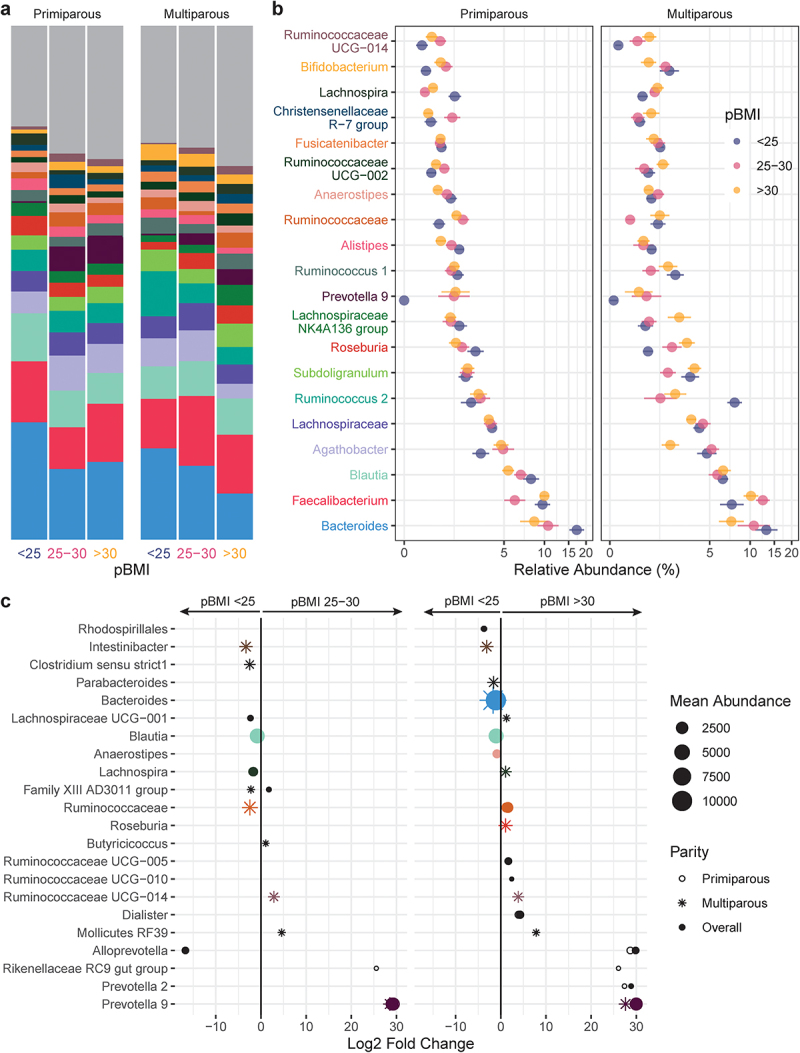
Taxonomic shifts with pBMI in pregnant gut microbiota. a-b, the relative abundances of the 20 most abundant genera in the maternal gut microbiota by pBMI < 25 (blue, *n* = 12), 25–30 (red, *n* = 10), and > 30 (yellow, *n* = 12) are shown as a, mean relative abundance in taxa bar plots and b, mean ± standard error of the mean for primiparous (pBMI <25, *n* = 7; 25–30, *n* = 6; >30, *n* = 7) and multiparous (pBMI <25, *n* = 5; 25–30, *n* = 4; >30, *n* = 5) participant with excess GWG. c, dot plots showing DESeq2 results of differentially abundant genera (mean abundance 150 reads) by pBMI category overall (filled dots) and in primiparous (no fill dots) and multiparous (stars) participants (dot size based on mean abundance, colors correspond to genus color in a where a differentially abundant genus is among the 20 most abundant genera).

Taxa that were differentially abundant during pregnancy between pBMI and GWG categories were identified using DESeq2 (RRID: SCR_015687). Overall, 14 genera with a mean abundance > 150 reads differed by pBMI category (Figure 3A–C, Supplemental Table S7) compared to participants with a pBMI < 25. Participants with a pBMI 25–30 had 6 differentially abundant genera, whereas participants with a pBMI > 30 had 11 differentially abundant genera (3 genera were differentially abundant for pBMI of both 25–30 and > 30). The relative abundance of *Blautia* was decreased in participants with a pBMI 25–30 or > 30 (Figure 3A–C). This was accompanied by decreased *Bacteroides* and *Anaerostipes* in participants with pBMI > 30. In contrast, *Prevotella 9* was markedly increased in participants with pBMI > 25 or > 30. In primiparous participants, a pBMI of 25–30 or > 30 was associated with increased *Rikenellaceae RC9 gut group*, which has been linked to higher propionate levels in high-fat fed mice,^29^ consistent with the increase observed in fecal propionate in participants with pBMI > 25 and/or excess GWG.

**Figure 4.**
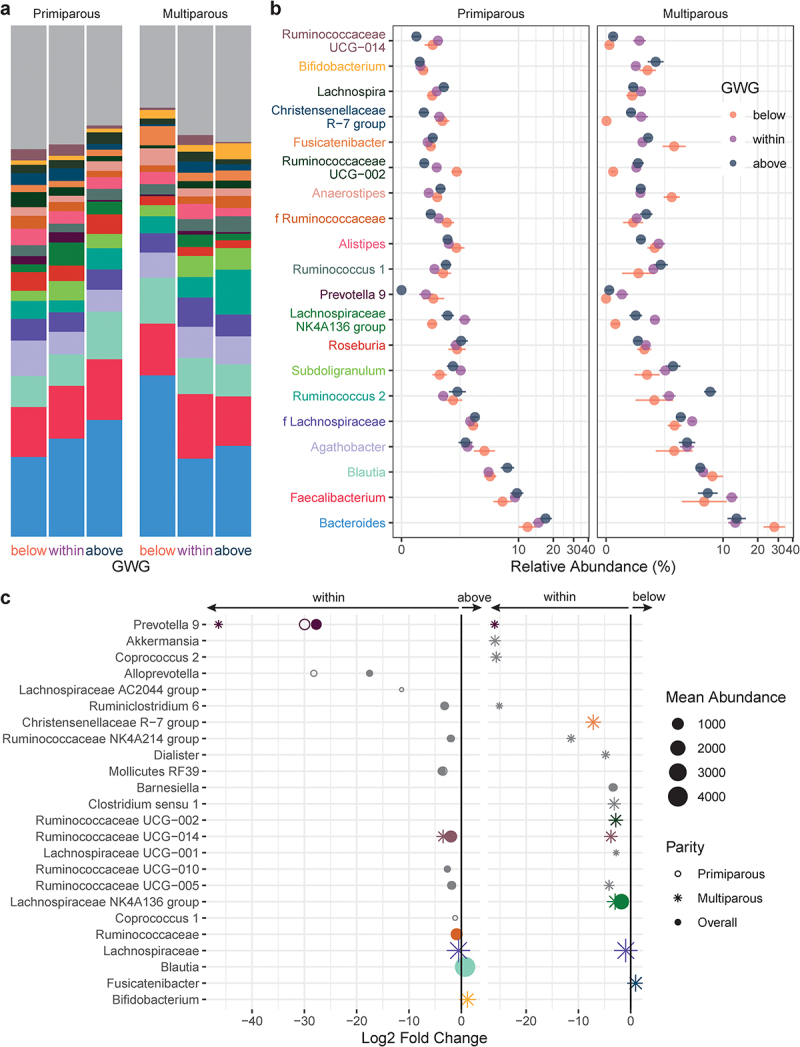
Taxonomic shifts with GWG in pregnant gut microbiota. a-b, the relative abundances of the 20 most abundant genera in the maternal gut microbiota by GWG below (orange, *n* = 7), within (purple, *n* = 17), and above (dark blue, *n* = 12) the recommended range are shown as a, mean relative abundance in taxa bar plots and b, mean ± standard error of the mean for primiparous (below, *n* = 5; within, *n* = 11; above, *n* = 7) and multiparous (below, *n* = 2; within, *n* = 6; above, *n* = 5) participant with pBMI < 25. c, dot plots showing DESeq2 results of differentially abundant genera (mean abundance > 150 reads) by GWG category overall (filled dots) and in primiparous (no fill dots) and multiparous (stars) participants (dot size based on mean abundance, colors correspond to genus color where a differentially abundant genus is among the 20 most abundant genera).

The relative abundances of 13 genera with mean abundance > 150 reads differed by GWG category (Figure 4A–C, Supplemental Table S7): compared to participants with appropriate GWG, participants with excess GWG (above) had 11 genera and participants with insufficient GWG (below) had 2 genera that were differentially abundant overall. *Prevotella 9* was markedly decreased by excess GWG, while insufficient GWG (below) was associated with decreased *Lachnospiraceae NK4A136 group*, the abundance of which has previously been associated with gut barrier function in mice.^30^ Excess GWG was also associated with decreased relative abundances of 6 genera of the SCFA-producing family Ruminococcaceae (Figure 4C), including uncultured genus (UCG) 005 which has previously been reported to decrease with gestational diabetes mellitus.^31^ In primiparous participants, excess GWG (above) was associated with a decrease in *Coprococcus 1*, which prior studies have found to be inversely associated with circulating triglyceride levels in nonpregnant individuals.^32^ In multiparous participants, excess GWG was associated with increased *Bifidobacterium*. Overall, *Bifidobacterium* was also increased in multiparous participants compared to primiparous participants (Supplemental Table S7).

### Maternal GWG and infant gut microbiota at 6 months of age

Of the 63 total mother-infant dyads who provided an infant fecal sample at 6 months of age, we excluded 3 for nicotine exposure during pregnancy, 1 for preterm birth, and 1 for low sequencing read count, which left 58 infants for full analysis. The majority of infants delivered by C-section were exposed to perinatal antibiotics (12/14; 86%;) while relatively few vaginally delivered infants were exposed (9/44; 20%). As perinatal antibiotic exposure has previously been shown to disturb vertical (maternal-to-infant) transmission of microbiota during birth,^33^ we assessed the impact of perinatal antibiotics (between 33 weeks gestation and birth, including antibiotics given during delivery) on the infant gut microbiota in this cohort. There was no significant impact of antibiotics on alpha diversity (Observed *p* = .70, Shannon *p* = .39; Supplemental Figure S3A). Infant gut microbiota beta diversity differed by antibiotic exposure in those born to primiparous participants (PERMANOVA, R^2^ = .068, *p* = .019), but not in those born to multiparous participants (R^2^ = 0.043, *p* = .56; Supplemental Figure S3B). This may reflect a rescue effect due to horizontal transmission from older siblings.^34^ Overall, perinatal antibiotic exposure was associated with a decreased abundance of 8 genera, including *Bifidobacterium*, *Streptococcus*, *Blautia*, and *Parabacteroides* (Supplemental Figure S4 A-C). Three additional genera (*Lachnoclostridium*, *Flavonifractor*, *Intestinibacter*) were significantly decreased by antibiotic exposure only in primiparous participants. *Akkermansia* and an unidentified genus of the family Lachnospiraceae were the only genera that increased with antibiotic exposure, although only in primiparous participants. In contrast, both these genera were decreased by antibiotic exposure in multiparous participants. These data are consistent with others that show that perinatal antibiotic exposure significantly perturbs the infant gut microbiota^33^ but extend these observations to show that parity influences these effects.

In infants that were not exposed to antibiotics (female, *n* = 19; male, *n* = 18) the majority were delivered vaginally (35/37; 95%). Infant characteristics at birth did not differ by maternal pBMI category overall or within primiparous and multiparous participants (Table 2). Although infant birth weight did not differ by GWG overall, excess GWG was associated with higher birth weight in multiparous participants (*p* = .028; Table 2), although this difference was not significant after adjusting for multiple testing. Overall, at 6 months of age, infant weight did not differ significantly by maternal pBMI, GWG, or parity. Among infants with available feeding data, at 6 months of age the majority were both breastfed (27/30; 90%) and eating solid foods (23/28; 82%) and these rates did not differ between groups (Table 2).

**Table 2.** Infant characteristics.

	Parity				Overall GWG				Primiparous GWG				Multiparous GWG			
Characteristic	primiparous^1^	multiparous^1^	p^2^	q^3^	within^1^	above^1^	p^2^	q^3^	within^1^	above^1^	p^2^	q^3^	within^1^	above^1^	p^2^	q^3^
n	24	13			12	20			7	12			5	8		
Maternal age	3.4 (4.3)	32.7 (3.5)	.073	0.3	32.0 (3.6)	31.1 (4.0)	.5	>0.9	30.6 (3.7)	30.7 (4.2)	.9	>0.9	34.0 (2.4)	31.9 (3.9)	.3	0.5
pBMI	24.2 (2.7, 29.1)	23.5 (21.5, 25.9)	.6	0.7	21.4 (18.6, 23.6)	25.8 (24.2, 30.5)	.002	0.011	18.7 (18.6, 25.4)	25.8 (24.2, 31.1)	.031	0.2	21.5 (21.3, 23.3)	25.0 (23.9, 27.7)	.019	0.076
GWG (kg)	15.0 (11.5, 19.0)	16.0 (14.0, 2.0)	.4	0.7	13.5 (13.0, 15.0)	19.0 (17.0, 20.2)	<.001	0.002	13.0 (12.0, 15.0)	19.0 (17.8, 20.2)	.003	0.036	14.0 (13.0, 15.0)	18.8 (16.8, 21.2)	.027	0.076
Length of gestation (days)	281 (276, 284)	280 (277, 284)	.8	0.8	282 (275, 282)	281 (278, 288)	.4	>0.9	282 (282, 283)	280 (276, 287)	.7	0.9	277.0 (270.0, 280.0)	283.0 (279.0, 289.0)	.078	0.14
Delivery mode = vaginal	22 (92%)	13 (100%)	.3	0.6	11 (92%)	19 (95%)	.7	>0.9	6 (86%)	11 (92%)	.7	0.9	5 (100%)	8 (100%)		
Infant sex = female	14 (58%)	5 (38%)	.3	0.6	5 (42%)	12 (60%)	.3	>0.9	4 (57%)	8 (67%)	.7	0.9	1 (20%)	4 (50%)	.3	0.5
Birth weight (g)	3,431 (447)	3,655 (353)	.11	0.3	3,540 (306)	3,526 (490)	>.9	>0.9	3,665 (246)	3,320 (510)	.069	0.3	3,366 (318)	3,835 (245)	.028	0.076
Birth length (cm)	51.83 (2.62)	51.38 (2.26)	.5	0.7	52.17 (2.48)	51.60 (2.58)	.6	>0.9	53.29 (2.75)	51.42 (2.54)	.2	0.3	50.60 (0.55)	51.88 (2.80)	.4	0.5
Birth weight:length	65 (61, 70)	72 (70, 74)	.024	0.3	68 (64, 71)	71 (61, 74)	.8	>0.9	69.0 (65.5, 71.6)	61.7 (59.9, 66.7)	.1	0.3	66.6 (64.7, 69.6)	72.9 (72.2, 74.9)	.023	0.076
Birth weight percentile	50 (20, 60)	61 (50, 80)	.073	0.3	57 (51, 62)	54 (20, 80)	.8	>0.9	59 (52, 62)	26 (12, 60)	.11	0.3	46 (28, 58)	72 (57, 82)	.062	0.14
Birth length percentile	46 (18, 74)	34 (23, 46)	.4	0.7	41 (30, 78)	46 (18, 62)	.5	>0.9	72 (42, 89)	49 (18, 68)	.3	0.5	31 (24, 38)	38 (20, 52)	.6	0.6
Infant weight at 6 months (g)	7,730 (6,960, 8,250)	7,738 (7,632, 8,154)	.5	0.7	7,800 (7,305, 8,280)	7,815 (7,611, 8,200)	>.9	>0.9	7,730 (6,960, 8,450)	7,940 (7,634, 8,200)	>.9	>0.9	7,959 (7,752, 8,154)	7,662 (7,598, 8,342)	.6	0.6

^1^n; Mean (SD); n (%); Median (IQR). ^2^Kruskal-Wallis rank sum test. ^3^False discovery rate correction for multiple testing.

**Figure 5.**
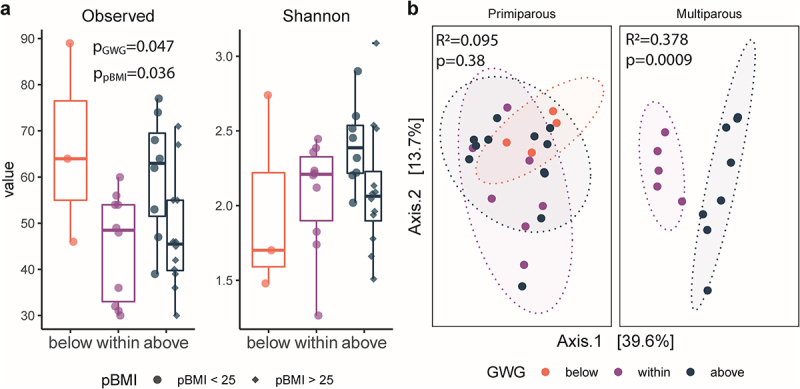
Parity modulates impact of GWG on infant gut microbiota. a, box plots of alpha diversity by gestational weight gain (GWG) category in primiparous participants (*n* = 24) and multiparous participants (*n* = 13). Alpha diversity (observed ASVs) was significantly increased by GWG above the recommended range (*p* = .034) in infants of participants with pBMI < 25. Significance assessed by a linear model (Kenward – Roger d.F.), with GWG category as a fixed effect. The box plot center line represents the median; the box limits represent the upper and lower quartiles; the whiskers represent the 1.5× interquartile range; the points represent the outliers. b, PCoA of Bray-Curtis distances shows clustering by maternal GWG category in infants of multiparous participants (R2 = 0.378, *p* = .0009) but not in infants of primiparous participants (R2 = 0.095, *p* = .38)). Primiparous (below, *n* = 4; within, *n* = 7; above = 12); multiparous (within, *n* = 5; above, *n* = 8). Significance was assessed by PERMANOVA.

We evaluated the impacts of maternal pBMI and GWG on the gut microbiota of infants at 6 months of age. We identified 1063 ASVs after removal of non-bacterial reads (Kingdom Eukaryota, Order Chloroplast, Family Mitochondria, or no assigned Phylum). The median sample read count was 59,561 (minimum 6,045; maximum 124,303). Overall, infant microbiota alpha diversity (number of observed ASVs) differed by maternal pBMI (*p* = .036) and GWG category (*p* = .047; Figure 5A) but did not differ by infant sex (*p* = .78). There was a significant effect of maternal GWG on beta diversity in infants of multiparous participants (PERMANOVA; R^2^ = 0.378, *p* = .0009), but not in infants of primiparous participants (R^2^ = 0.0950, *p* = .377; Figure 5B). Infant beta diversity did not differ by maternal pBMI category (R^2^ = 0.0511, *p* = .565) or infant sex (R^2^ = 0.0126, *p* = .914). Therefore, in addition to previous reports linking maternal GWG and BMI to shifts in the infant gut microbiota,^19,35–40^ we now show that parity modulates the impact of maternal GWG on the infant gut microbiota.

**Figure 6.**
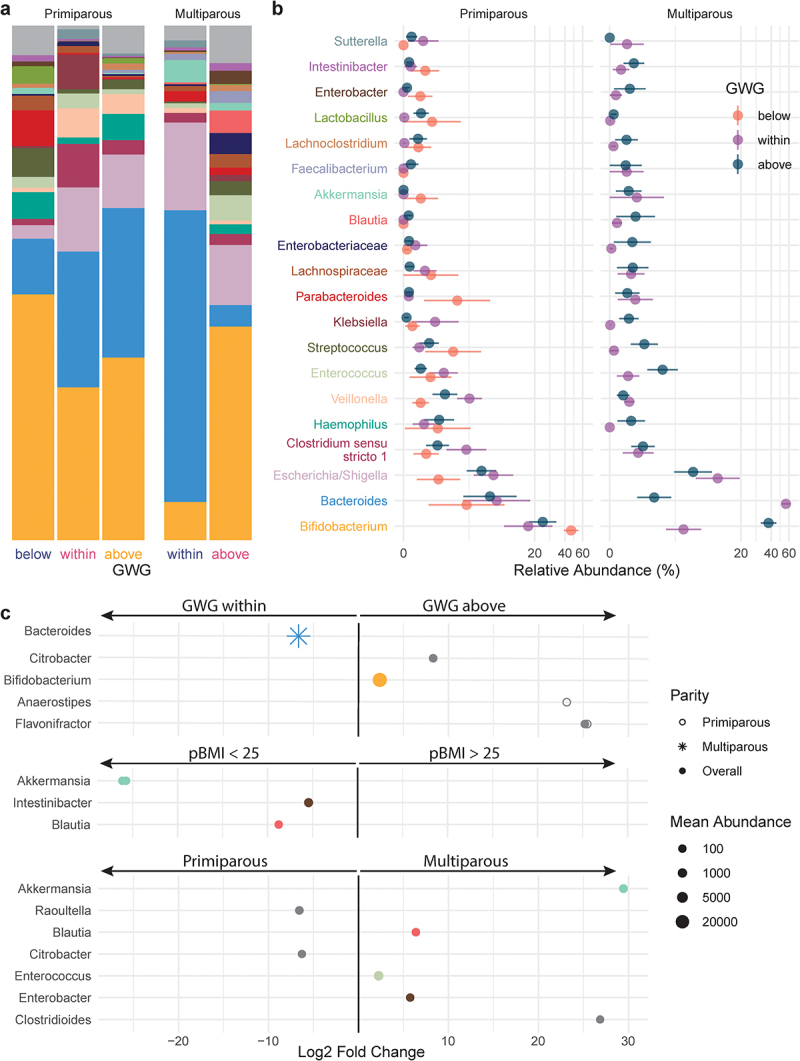
Taxonomic shifts with maternal GWG in infant gut microbiota. a-b, the relative abundances of the 20 most abundant genera in the infant gut microbiota by GWG below (orange), within (purple), and above (dark blue) the recommended range are shown as a, mean relative abundance in taxa bar plots and b, mean ± standard error of the mean for infants of primiparous (below, *n* = 4; within, *n* = 7; above, *n* = 12) and multiparous (within, *n* = 5; above, *n* = 8) participants. c, dot plots showing DESeq2 results of differentially abundant genera overall (filled dots) and in primiparous (no fill dots) and multiparous (stars) participants by maternal GWG, BMI, and parity. Dot size based on mean abundance, colors correspond to genus color in a where a differentially abundant genus is among the 20 most abundant genera.

Taxa that were differentially abundant in infants between maternal pBMI and GWG categories were identified using DESeq2. Overall, maternal excess GWG (above) was associated with an increased abundance of *Bifidobacterium* (Figure 6A–C) – echoing similar shifts in the maternal gut microbiota (Figure 4A–C). In infants of multiparous but not primiparous participants, this was accompanied by a decrease in *Bacteroides*. Compared to a maternal pBMI 18.5–25, a maternal pBMI > 25 was associated with a decreased relative abundance of Clostridiales genera *Blautia* and *Intestinibacter* in the infant gut microbiota, which were also decreased by pBMI > 25 in the maternal gut microbiota. Depleted *Blautia* in obese children has been linked to intestinal inflammation,^41^ which is consistent with an observed association between excess maternal adiposity and childhood obesity and inflammation.^42^ This overlap in differentially abundant taxa between the maternal and infant gut microbiota is consistent with vertical transmission of the maternal gut microbiota.^43^

## Discussion

Pregnancy-associated shifts in maternal gut microbiota have been implicated in mediating metabolic adaptations to pregnancy,^4^ but few studies have investigated the gut microbiota longitudinally over the course of healthy pregnancy. Because of this, there exists contrasting data regarding whether these temporal shifts do^4^ or do not^6,44^ occur. In this study, we investigated the independent and combined impacts of maternal pre-pregnancy BMI (pBMI) and gestational weight gain (GWG) on maternal gut microbiota over the course of pregnancy and lactation and infant gut microbiota at 6 months of age. We show that pregnancy-associated remodeling of the gut microbiota is individual-specific and therefore longitudinal sampling within individuals is essential. We found that pBMI and GWG impact the maternal gut microbiome during pregnancy – both the microbial composition and possible function via SCFA metabolites – and also impact the infant gut microbiota at 6 months of age. We also show for the first time in humans that parity modulates the impact of pBMI on pregnancy-associated remodeling of the maternal gut microbiota and also modulates the impact of perinatal antibiotic exposure and GWG on the infant gut microbiota. We now suggest that the maternal gut microbiota may retain a “ecological memory”^45^ of previous pregnancies.

We found that in primiparous (first) pregnancy, the gut microbiota of participants with higher pBMI changed less over the course of pregnancy and postpartum than that of participants with lower pBMI. Previous studies of the gut microbiota have found little-to-no overall effect of pBMI on overall beta diversity in the first trimester^46^ and across pregnancy^5,44^ but have not investigated beta diversity within individuals over the course of pregnancy. Shifts in the gut microbiome due to diet or antibiotic perturbation are highly individual-specific,^47^ and our data now suggest that pregnancy-associated remodeling of the gut microbiota is also individual-specific. Our observed decrease in intraindividual beta diversity with increasing pBMI in primiparous participants is consistent with a decreased magnitude of metabolic adaptation to pregnancy with increasing pBMI^48–50^ and highlights the necessity of longitudinal sampling for investigations of pregnancy-associated microbiota remodeling.

Our novel observation that this remodeling was not associated with pBMI in multiparous participants suggests that microbiotal adaptations to first pregnancy may have persistent impacts that modulate microbiotal adaptations to subsequent pregnancies. The impact of parity on the gut microbiome and its contribution to metabolic adaptations to pregnancy has not been previously investigated in human pregnancy. Recently, Berry et al. found that maternal microbiotal adaptations to pregnancy occurred more rapidly with increasing parity in pigs.^51^ This may reflect increased adaptive plasticity resulting from adaptations to first pregnancy.^52^ Indeed, first-pregnancies are associated with greater maternal constraint^12,53^ compared to subsequent pregnancies, which may include reduced or delayed metabolic adaptations to pregnancy.^23^ Whether a functional ecological memory exists from one pregnancy to the next in humans is still unclear, but our data support the notion that parity influences microbial community composition and must be considered when investigating human pregnancy-related microbiotal (and intestinal) adaptations.

Maternal adaptations to pregnancy include increased intestinal surface area and increased transit time, allowing increased nutrient uptake to provide the energy required by fetal development and lactation.^54^ SCFAs may support these intestinal adaptations by promoting proliferation of intestinal epithelium^55^ and improving intestinal barrier function.^27,56^ Although human data on intestinal adaptations to pregnancy are limited, in animal models we have found that excess adiposity during pregnancy is associated with decreased intestinal length and increased intestinal permeability.^57^ In this study, we found that parity modulated the impact of pBMI and GWG on the relative abundance of maternal fecal SCFAs. In primiparous participants, excess GWG was associated with decreased acetate and increased propionate. As fecal SCFAs levels may be more strongly correlated to intestinal absorption than production,^58^ these changes could be due to altered epithelial cell uptake resulting from reduced intestinal adaptations to pregnancy, but this hypothesis remains to be tested.

Alternatively, differences in fecal SCFA levels may result from shifts in microbial production due to altered microbiota community composition. We found increased propionate in primiparous participants with excess GWG and/or pBMI > 25, consistent with a prior study that found increased fecal propionate in pregnant individuals with a pBMI > 25 compared to pBMI < 25, although parity and GWG were not reported.^59^ *Prevotella*-dominant microbiota produce more propionate – and more SCFAs overall – than *Bacteroides*-dominant microbiota.^60^ This is consistent with our finding that *Prevotella* was increased in participants with a pBMI > 25, while *Bacteroides* was decreased, particularly in primiparous participants. A previous study also found decreased *Bacteroides* in the first and third trimesters with pre-pregnancy overweight,^17^ although others have reported increased levels in the third trimester with pre-pregnancy overweight but not obesity.^18,61^ In independent studies, *Prevotella* has been associated with improved glucose tolerance^62^ and impaired glucose tolerance.^63^ This functional diversity mirrors that of *Bacteroides* and suggests that the impacts of these genera depend on host physiology, including pregnancy history. Future studies are needed to longitudinally investigate pregnancies within participants as well as in the intrapartum period to test this hypothesis.

Shifts in the maternal gut microbiota can impact the infant gut microbiome by (1) altering fetal intestinal development through the *in utero* environment and (2) direct transmission of maternal microbes to the infant during and after birth. Disruption of this vertical transmission may have persistent effects on the infant gut microbiota, as we found perinatal antibiotic exposure was associated with a decrease in *Bifidobacterium* in the infant gut microbiota at 6 months of age. We show novel data that support the position that parity modulates the impact of maternal GWG on infant characteristics at birth and the infant gut microbiota at 6 months of age. Excess GWG is often associated with a reduced risk of small for gestational age (SGA) birth weight and an increased risk of large for gestational age (LGA) birth weight,^64^ although some data suggest that maternal obesity can also be associated with intrauterine growth restriction (IUGR).^65^ Little to no data exist on the modulating effect of parity. We found that excess GWG was associated with higher birth weight in infants of multiparous participants, and a tendency toward lower birth weight in infants of primiparous participants. These impacts of excess GWG were also associated with a shift in microbiota beta diversity at 6 months of age in infants of multiparous – but not primiparous – participants. These same infants also had a decrease in *Bacteroides* and an increase in *Bifidobacterium* reflecting similar shifts in the maternal gut microbiota consistent with the high rates of vertical transmission previously found for these genera.^66^ Therefore, health behaviors during pregnancy and the postpartum/intrapartum interval should be supported not only for maternal health but also infant microbiotal health.

Our study has both strengths and limitations. Our longitudinal sampling of a prospective pregnancy cohort allowed us to explore beta diversity within participants to overcome the overwhelming effect of interindividual variation. However, as we recruited participants in the first trimester of pregnancy, we are unable to address pregnancy-associated shifts in the gut microbiota that may occur very early in gestation. We also show potential functional outcomes of observed microbial shifts in our analysis of fecal SCFA levels, but did not quantify circulating SCFA levels, which may more directly modulate host metabolism.^67^ We also cannot measure whether production versus absorption on these SCFAs is altered with pregnancy, GWG, BMI, or parity. Functional analyses using metagenomics would significantly contribute to these observations and the field of perinatal microbial profiling. We acknowledge that our sample size is limited but despite this we should robust relationships and suggest that future studies must consider parity in their experimental design when investigating maternal microbial profiling. Finally, although our study is unique among studies of the pregnancy microbiome in its design and relationship to parity, we were unable to examine any interacting effects of pBMI and GWG as the vast majority of participants with high pBMI also had excess GWG.

In conclusion, we suggest that parity modulates the impact of pBMI on pregnancy-associated remodeling of the maternal gut microbiota, colonization of the neonatal gut, and the impact of GWG on the infant gut microbiota. We also show that in women in their first pregnancy, high pBMI is associated with diminished remodeling of the gut microbiome during pregnancy, which may reflect a reduced requirement for metabolic adaptations to pregnancy. This is accompanied by functional impacts via altered SCFA levels, which may reflect reduced intestinal adaptations to pregnancy. These outcomes are important not only to maternal health, but also to infant health as infants born to multiparous women with excess gestational weight gain show distinct shifts in their microbiota. Finally, our data suggest that the human gut microbiome may retain an “ecological memory”^45^ of prior pregnancies^51^ which may serve as an adaptation that minimizes maternal constraint in subsequent pregnancies. These data must be considered when designing therapeutic strategies involving the maternal and infant microbiome and may serve to reduce intraindividual variability in these therapeutic studies.

## Methods

### Data and code availability

Sequencing data have been deposited at the NCBI Sequence Read Archive (SRA; PRJNA878704) and are publicly available as of the date of publication. All original code has been deposited on GitHub (https://github.com/kennek6/Kennedyetal2023/) and is publicly available as of the date of publication. Any additional information required to reanalyze the data reported in this paper is available from the lead contact upon request.

### Study design

The study protocol was reviewed and approved by the Charité ethics committee (EA 4/059/16). All participants provided written informed consent. Healthy females >18 years of age with singleton pregnancies were prospectively recruited at an urban clinic between 9‐17 weeks of gestation at the Charité University Berlin and obstetrical history was collected by an obstetrician. We excluded participants who (1) had severe chronic gastrointestinal diseases or conditions, (2) had any significant heart, kidney, liver, or pancreatic diseases, (3) had pre‐existing diabetes, (4) had depression, or (5) delivered infants with severe malformations noted at birth. A total of 89 participants were included in the study. We additionally excluded maternal and infant samples from participants with nicotine exposure during pregnancy and excluded maternal samples from participants who took antibiotics during pregnancy. After these exclusions, this study included infant samples from 58 participants, and maternal samples from 65 participants (Supplemental Figure S5).

### Sample collection

Participants were evaluated at the end of the first trimester (8‐18 weeks gestation), second trimester (25–29 weeks), and third trimester (29–38 weeks) of pregnancy, at delivery (first bowel movement following delivery), and at 6 months postpartum. Pre-pregnancy body mass index (pBMI) was obtained from measured height recruitment and recalled pre-pregnancy weight, which was compared to BMI measures taken at the first study visit to validate accuracy. Gestational weight gain (GWG) was calculated from measured weight at delivery and recalled pre-pregnancy weight. At each study visit, participant weight was measured and participants completed a 25-item food frequency questionnaire (FFQ)^68^ adapted from the validated PrimeScreen FFQ.^24^ Participants collected fecal samples at home prior to each study visit following a standardized sterile stool collection protocol. Stool was frozen at −20°C immediately after collection in the provided sample collection bags and stored at −80°C upon arrival. None had taken prebiotics, laxatives, or diarrhea inhibitors in the days before sampling. Samples were kept on dry-ice during aliquoting for further analyses to avoid freeze-thaw cycles.

### DNA extraction and amplification

Genomic DNA (gDNA) was extracted from fecal samples as described previously^69^ on a MagMAX Express semi-automatic robot (MagMAX-96 DNA Multi-Sample Kit; Invitrogen, cat# 4413022) with the addition of a mechanical lysis step using 0.2 g of 2.8 mm ceramic beads to improve extraction efficiency and without mutanolysin. Four negative controls were included on each extraction plate. PCR amplification of the variable 3 and 4 (V3-V4) regions of the 16S rRNA gene was performed on the extracted DNA using methods previously described.^70^ Each reaction contained 5 pmol of primer (341F – CCTACGGGNGGCWGCAG, 806 R – GGACTACNVGGGTWTC-TAAT), 200 mM of dNTPs, 1.5 μl 50 mM MgCl2, 2 μl of 10 mg/ml bovine serum albumin (irradiated with a transilluminator to eliminate contaminating DNA) and 0.25 μl Taq polymerase (Life Technologies, Canada) for a total reaction volume of 50 μl. 341F and 806 R rRNA gene primers were modified to include adapter sequences specific to the Illumina technology and 6-base pair barcodes were used to allow multiplexing of samples. Lack of amplification in all negative controls was confirmed by gel electrophoresis and one negative control from each extraction plate was sequenced.

### 16S rRNA gene sequencing

16S DNA products of PCR amplification were sequenced using the Illumina MiSeq platform (2×300bp) at the Farncombe Genomics Facility (McMaster University, Hamilton ON, Canada). Primers were trimmed from FASTQ files using Cutadapt^71^ (RRID: SCR_011841) and DADA2^72^ was used to derive amplicon sequence variants (ASVs). Taxonomy was assigned using the Silva 132 reference database.^73^ Non-bacterial ASVs were culled (kingdom Eukaryota, family Mitochondria, order Chloroplast, or no assigned phylum), as was any ASV to which only one sequence was assigned. No additional prevalence- or abundance-based filtering was performed.

### Quantification of short-chain fatty acids

SCFA levels were measured in a subset of fecal samples by gas chromatography – mass spectroscopy (GC-MS) at the McMaster Regional Centre of Mass Spectrometry. A weight equivalent amount of 3.7% HCl, 10 µL of internal standard, and 500 µL of diethyl ether was added to each fecal sample and vortexed for 15 min. After vortexing, 400 µL of diethyl ether fecal extract was transferred to a clean 1.5 mL Eppendorf tube. In a chromatographic vial containing an insert, 20 µL of N-tert-butyldimethylsilyl-N-methyltrifluoroacetamide was added, after which 60 µL of diethyl ether fecal extract was added. The mixture was incubated at room temperature for one hour and analyzed using GC-MS (6890N GC, coupled to 5873N Mass Selective Detector; Agilent Technologies, Santa Clara, CA, USA). Statistical significance was assessed by linear mixed model with pBMI, GWG category, and parity as fixed effects and participant ID as a random effect, and multiple comparisons by pBMI and GWG category were performed within primiparous and multiparous participants (Kenward-Rogers degrees of freedom with Tukey adjustment for multiple comparisons).

### Statistical analysis

#### Participant characteristics

Participant characteristic and diet variables (Tables 1 and 2, Supplemental Tables S1-S3) were analyzed in R using the gtsummary package (RRID: SCR_021319). Normality was determined by Shapiro-Wilk test (rstatix; RRID: SCR_021240) and significance was assessed by Kruskal-Wallis test and false discovery rate correction for multiple testing.

#### 16S analysis

We performed alpha and beta diversity analyses in R using phyloseq^74^ (RRID: SCR_013080). Alpha diversity analyses were performed on rarefied data (14872 reads per sample). Significance of alpha diversity was analyzed by linear mixed model (Kenward – Roger degrees of freedom; lme4 RRID: SCR_015654) with time point, pBMI, or GWG category as a fixed effect and participant ID as a random effect. Beta diversity analyses were performed on proportionally normalized data. These results were visualized via Principal Coordinate Analysis (PCoA) ordination using R’s ggplot2 package (RRID: SCR_014601).^75^ We tested for whole community differences across groups using vegan’s^76^ (RRID: SCR_011950) implementation of permutational multivariate analysis of variance (PERMANOVA) in the adonis2 command blocked by sample time point as strata to account for repeated measures. Significance of Bray-Curtis dissimilarity was analyzed within primiparous and multiparous participants by linear mixed model with pBMI, GWG category, and sample time point as interacting fixed effects and participant ID as a random effect. Differential abundance analysis was performed using DESeq2 (RRID: SCR_015687) across all pregnancy time points. We did not assess interacting effects of pBMI and GWG as (1) pBMI category and GWG category were collinear (χ-squared *p* < .0001) and (2) all but four participants with a pBMI > 25 experienced excess GWG. These four participants were excluded from differential abundance analyses and the impact of pBMI was assessed within participants with excess GWG (to control for the effect of GWG) and the impact of GWG was assessed in participants with a pBMI 18.5–25 (to control for the effect of pBMI).

#### SCFA analysis

Statistical significance was assessed by linear mixed models with pBMI category and parity or GWG category and parity as fixed effects and participant ID as a random effect, and multiple comparisons by pBMI and GWG category were performed within primiparous and multiparous participants (Kenward-Rogers degrees of freedom with Tukey adjustment for multiple comparisons). All statistical details, including statistical tests used, sample sizes, and definition of center and dispersion can be found in the figure legends.

## Supplementary Material

Supplemental Material
