# Supplementary material for: Parity modulates impact of BMI and gestational weight gain on gut microbiota in human pregnancy
Source: Gut Microbes. 2023 Oct 9;15(2):2259316. doi: 10.1080/19490976.2023.2259316 (PMC10563629; doi:10.1080/19490976.2023.2259316)
Supplement: Supplemental Material [file KGMI_A_2259316_SM6322.zip › Supplementary files/TableS1clean.docx]

**Supplemental Table 1. Impact of pBMI on diet**

First trimester

Second trimester

Third trimester

Weekly servings

**pBMI<25***^1^*

**25-30***1*

**>25***1*

**p***2*

**q***3*

**pBMI<25***^1^*

**25-30***1*

**>25***1*

**p***2*

**q***3*

**pBMI<25***^1^*

**25-30***1*

**>25***1*

**p***2*

**q***3*

n

25

7

11

20

8

10

21

8

12

Green vegetables

1.52 (3.65)

0.86 (0.24)

0.91 (0.20)

*0.56*

*0.92*

1.75 (4.07)

0.69 (0.26)

2.70 (5.73)

*0.13*

*0.54*

0.90 (0.20)

0.81 (0.26)

2.21 (5.29)

*0.17*

*0.93*

Carrots

2.96 (4.94)

2.07 (1.17)

2.00 (1.16)

*0.78*

*0.96*

2.05 (1.09)

1.94 (1.15)

1.85 (1.23)

*0.80*

*0.87*

2.83 (3.86)

2.19 (1.13)

1.96 (1.10)

*0.92*

*0.93*

Citrus

2.3 (3.6)

1.4 (1.1)

4.6 (7.2)

*0.86*

*0.96*

4.0 (6.5)

5.4 (8.4)

7.0 (8.4)

*0.37*

*0.80*

4.6 (7.2)

3.6 (6.3)

6.2 (7.8)

*0.42*

*0.93*

Whole dairy

9.0 (8.4)

9.6 (8.8)

10.9 (9.3)

*0.96*

*0.96*

10.7 (8.6)

14.8 (7.9)

14.0 (8.1)

*0.43*

*0.80*

7.2 (7.7)

8.8 (8.5)

10.3 (9.1)

*0.88*

*0.93*

Low-fat greek yogurt

0.60 (0.20)

0.50 (0.00)

0.59 (0.20)

*0.45*

*0.83*

0.73 (0.57)

0.56 (0.18)

0.60 (0.21)

*0.74*

*0.84*

1.60 (4.03)

0.50 (0.00)

0.58 (0.19)

*0.21*

*0.93*

Cottage cheese

1.40 (3.70)

0.50 (0.00)

0.86 (0.74)

*0.21*

*0.54*

0.98 (0.90)

0.56 (0.18)

0.75 (0.79)

*0.25*

*0.71*

1.02 (0.99)

0.81 (0.88)

0.92 (0.97)

*0.64*

*0.93*

Eggs

1.86 (1.13)

2.07 (1.17)

1.27 (0.88)

*0.36*

*0.72*

1.63 (1.05)

1.44 (0.98)

1.40 (0.84)

*0.94*

*0.94*

1.55 (0.95)

1.44 (0.98)

1.50 (1.13)

*0.69*

*0.93*

Nuts

3.1 (4.9)

1.4 (1.1)

5.0 (7.0)

*0.25*

*0.57*

4.3 (6.4)

1.6 (1.2)

2.4 (1.1)

*0.55*

*0.80*

4.8 (7.1)

1.5 (1.3)

3.1 (5.1)

*0.62*

*0.93*

Processed meat

1.24 (1.03)

1.14 (0.85)

1.32 (1.10)

*0.92*

*0.96*

1.60 (1.19)

1.19 (1.13)

1.40 (1.13)

*0.60*

*0.81*

1.81 (1.18)

1.25 (1.10)

1.67 (1.19)

*0.48*

*0.93*

Seafood

1.20 (0.95)

1.21 (0.81)

1.00 (0.71)

*0.67*

*0.96*

0.93 (0.54)

0.75 (0.27)

1.50 (1.05)

*0.15*

*0.54*

0.76 (0.26)

0.75 (0.27)

1.00 (0.67)

*0.54*

*0.93*

Whole grains

9.0 (8.3)

11.9 (8.9)

7.1 (7.7)

*0.64*

*0.96*

7.7 (7.6)

10.5 (9.1)

7.4 (8.0)

*0.83*

*0.87*

8.2 (7.8)

8.8 (8.5)

8.0 (8.2)

*0.91*

*0.93*

Sweetened drinks

2.36 (3.65)

0.93 (0.93)

2.95 (5.43)

*0.20*

*0.54*

3.1 (5.5)

1.3 (1.1)

2.6 (5.8)

*0.75*

*0.84*

1.57 (1.16)

1.31 (1.07)

2.83 (5.19)

*0.91*

*0.93*

Salt

3.4 (6.0)

3.9 (6.8)

1.0 (1.0)

*0.13*

*0.49*

3.15 (5.53)

1.50 (1.25)

1.05 (1.04)

*0.48*

*0.80*

2.02 (4.03)

1.44 (1.29)

0.96 (0.96)

*0.67*

*0.93*

*1* N; Mean (SD)

*2* Kruskal-Wallis rank sum test

*3* False discovery rate correction for multiple testing

Fried foods 0.68 (0.24) 0.50 (0.00) 0.91 (0.74) *0.11 0.48* 0.80 (0.57) 0.63 (0.23) 0.70 (0.26) *0.72 0.84* 0.76 (0.56) 0.56 (0.18) 0.92 (0.70) *0.22 0.93*

Baked goods 2.28 (3.63) 1.36 (1.14) 2.73 (5.48) *0.26 0.57* 2.53 (4.01) 2.75 (0.71) 2.15 (1.11) *0.12 0.54* 3.00 (3.79) 2.12 (1.22) 3.83 (4.86) *0.53 0.93*

Refined grains 4.2 (5.7) 1.4 (1.1) 1.2 (0.9) *0.009* *0.24* 4.8 (6.2) 1.7 (1.1) 1.6 (1.2) *0.080 0.52* 3.4 (5.3) 1.8 (1.3) 4.7 (6.8) *0.58 0.93*

Poultry 1.30 (1.00) 1.00 (0.91) 1.91 (1.04) *0.037* *0.42* 1.30 (1.03) 3.62 (6.29) 1.70 (1.14) *0.44 0.80* 1.26 (1.02) 1.69 (1.10) 2.12 (1.09) *0.051 0.67*

Red meat 1.40 (1.04) 1.71 (1.22) 1.41 (1.04) *0.86 0.96* 1.23 (0.94) 2.25 (1.04) 2.15 (1.11) *0.017* *0.44* 1.40 (1.06) 1.06 (0.82) 1.71 (1.16) *0.51 0.93*

Legumes 1.00 (0.79) 1.07 (0.89) 0.77 (0.26) *0.93 0.96* 1.28 (0.91) 0.63 (0.23) 1.05 (0.72) *0.040* *0.51* 0.88 (0.55) 0.81 (0.26) 0.75 (0.26) *0.83 0.93*

Plant milks 0.70 (0.69) 1.57 (1.34) 0.55 (0.15) *0.048* *0.42* 0.65 (0.56) 0.81 (0.88) 0.50 (0.00) *0.55 0.80* 1.62 (4.05) 0.81 (0.88) 0.54 (0.14) *0.83 0.93*

Low-fat yogurt 2.14 (3.69) 1.43 (1.10) 3.00 (5.41) *0.94 0.96* 2.15 (4.10) 0.94 (0.86) 1.20 (0.98) *0.66 0.84* 3.33 (5.33) 0.63 (0.23) 1.17 (1.11) *0.018 0.47*

Low-fat milk 4.8 (7.3) 1.3 (1.2) 2.9 (5.5) *0.83 0.96* 2.5 (4.0) 3.8 (6.3) 6.8 (8.4) *0.53 0.80* 6.6 (8.1) 1.8 (1.3) 4.7 (6.8) *0.58 0.93*

Other fruits 13.9 (7.6) 9.9 (8.6) 13.2 (8.1) *0.48 0.84* 12.5 (8.2) 8.8 (8.5) 12.4 (8.5) *0.52 0.80* 12.0 (8.3) 8.8 (8.5) 10.8 (8.5) *0.61 0.93*

Other vegetables 7.4 (7.4) 5.0 (6.2) 13.0 (8.3) *0.11 0.48* 5.8 (6.8) 7.0 (7.4) 9.2 (8.5) *0.39 0.80* 4.3 (4.9) 2.8 (0.7) 6.8 (7.4) *0.37 0.93*

Crucifers 1.08 (0.89) 1.29 (0.76) 1.23 (0.90) *0.20 0.54* 1.25 (0.92) 1.13 (0.79) 0.65 (0.24) *0.059 0.51* 1.00 (0.71) 1.13 (0.79) 1.13 (0.91) *0.82 0.93*

Quality score 9.1 (4.0) 10.9 (2.5) 12.4 (4.3) *0.11 0.48* 9.5 (5.6) 7.1 (3.9) 10.7 (5.3) *0.23 0.71* 10.3 (5.6) 9.0 (3.5) 9.2 (4.9) *0.93 0.93*
