# Supplementary material for: Parity modulates impact of BMI and gestational weight gain on gut microbiota in human pregnancy
Source: Gut Microbes. 2023 Oct 9;15(2):2259316. doi: 10.1080/19490976.2023.2259316 (PMC10563629; doi:10.1080/19490976.2023.2259316)
Supplement: Supplemental Material [file KGMI_A_2259316_SM6322.zip › Supplementary files/TableS2clean.docx]

**Supplemental Table 2. Impact of gestational weight gain category on diet**

First trimester

Second trimester

Third trimester

**below***1*

**within***^1^*

**above***1*

**p***2*

**q***3*

**below***1*

**within***^1^*

**above***1*

**p***2*

**q***3*

**below***1*

**within***^1^*

**above***1*

**p***2*

**q***3*

Weekly servings

n

6

13

23

6

9

23

6

12

22

Green vegetables

0.83 (0.26)

0.92 (0.19)

1.57 (3.81)

*0.35*

*0.95*

3.83 (7.43)

0.89 (0.22)

1.59 (3.80)

*0.73*

*0.95*

1.00 (0.00)

0.88 (0.23)

1.59 (3.90)

*0.16*

*0.81*

Carrots

1.17 (0.93)

3.23 (4.86)

2.54 (3.76)

*0.32*

*0.95*

2.33 (1.03)

2.06 (1.13)

1.85 (1.14)

*0.53*

*0.86*

2.17 (1.29)

3.50 (4.98)

1.93 (1.11)

*0.64*

*0.93*

Citrus

1.5 (1.2)

3.1 (4.9)

2.9 (5.2)

*0.61*

*0.95*

6.8 (9.5)

3.6 (5.9)

5.2 (7.5)

*0.78*

*0.95*

7.0 (9.3)

5.6 (8.1)

4.2 (6.1)

*0.90*

*0.93*

Whole dairy

4.8 (7.0)

8.7 (8.5)

11.6 (8.7)

*0.25*

*0.95*

7.9 (8.6)

15.2 (7.5)

12.5 (8.3)

*0.28*

*0.86*

5.2 (6.8)

8.0 (8.2)

9.1 (8.5)

*0.76*

*0.93*

Low-fat greek yogurt

0.67 (0.26)

0.58 (0.19)

0.57 (0.17)

*0.50*

*0.95*

0.67 (0.26)

0.89 (0.82)

0.57 (0.17)

*0.30*

*0.86*

3.67 (7.51)

0.79 (0.72)

0.55 (0.15)

*0.24*

*0.81*

Cottage cheese

0.50 (0.00)

0.62 (0.22)

1.59 (3.86)

*0.37*

*0.95*

0.58 (0.20)

0.94 (0.81)

0.85 (0.86)

*0.33*

*0.86*

0.92 (1.02)

1.21 (1.10)

0.84 (0.88)

*0.23*

*0.81*

Eggs

1.83 (1.29)

1.85 (1.13)

1.70 (1.08)

*0.93*

*0.98*

2.00 (1.10)

1.56 (1.10)

1.39 (0.88)

*0.38*

*0.86*

2.00 (1.10)

1.50 (0.90)

1.41 (1.02)

*0.25*

*0.81*

Nuts

4.2 (7.3)

1.8 (1.2)

3.9 (6.1)

*0.92*

*0.98*

7.2 (9.2)

3.7 (5.9)

2.0 (1.2)

*0.94*

*0.95*

6.8 (9.5)

4.5 (6.9)

2.4 (3.9)

*0.97*

*0.97*

Processed meat

0.83 (0.26)

1.35 (1.16)

1.30 (1.05)

*0.94*

*0.98*

0.58 (0.20)

2.00 (1.20)

1.48 (1.15)

*0.046*

*0.60*

0.75 (0.27)

2.21 (1.18)

1.55 (1.14)

*0.10*

*0.81*

Seafood

1.08 (0.97)

0.92 (0.67)

1.30 (0.94)

*0.32*

*0.95*

0.75 (0.27)

1.06 (0.77)

1.11 (0.78)

*0.55*

*0.86*

0.75 (0.27)

0.79 (0.26)

0.89 (0.53)

*0.89*

*0.93*

Whole grains

8.0 (8.6)

8.8 (8.4)

9.7 (8.4)

*0.91*

*0.98*

10.7 (9.2)

6.6 (7.1)

8.2 (8.1)

*0.85*

*0.95*

8.3 (8.3)

6.8 (7.4)

8.5 (8.1)

*0.87*

*0.93*

Sweetened drinks

1.50 (1.18)

1.69 (1.27)

2.87 (5.20)

*>0.99*

*>0.99*

4.1 (7.4)

3.3 (6.0)

1.9 (3.8)

*0.79*

*0.95*

1.08 (0.97)

1.67 (1.19)

2.18 (3.90)

*0.63*

*0.93*

Salt

1.0 (1.0)

2.6 (5.0)

3.5 (6.2)

*0.70*

*0.96*

1.00 (1.00)

5.22 (7.88)

1.41 (1.19)

*0.46*

*0.86*

1.33 (1.29)

2.29 (5.31)

1.23 (1.12)

*0.85*

*0.93*

*1* N; Mean (SD)

*2* Kruskal-Wallis rank sum test

*3* False discovery rate correction for multiple testing

Fried foods 0.58 (0.20) 0.65 (0.24) 0.78 (0.54) *0.54 0.95* 1.00 (1.00) 0.72 (0.26) 0.67 (0.24) *0.88 0.95* 0.75 (0.27) 0.79 (0.72) 0.77 (0.55) *0.69 0.93*

Baked goods 3.75 (7.47) 1.77 (1.20) 2.17 (3.80) *0.62 0.95* 2.00 (1.10) 3.83 (5.79) 2.07 (1.10) *0.95 0.95* 2.00 (1.10) 4.00 (4.79) 2.95 (3.73) *0.28 0.82*

Refined grains 2.3 (1.0) 4.3 (6.6) 2.3 (3.8) *0.44 0.95* 4.7 (7.1) 6.0 (7.4) 1.8 (1.1) *0.25 0.86* 3.8 (7.4) 3.2 (5.1) 3.6 (5.1) *0.46 0.93*

Poultry 1.17 (0.93) 1.58 (1.19) 1.41 (0.98) *0.84 0.98* 1.08 (0.97) 1.50 (1.15) 2.26 (3.80) *0.50 0.86* 1.17 (0.93) 1.63 (1.23) 1.73 (1.10) *0.51 0.93*

Red meat 0.67 (0.26) 1.65 (1.13) 1.59 (1.07) *0.065 0.85* 1.17 (0.93) 1.06 (0.77) 2.07 (1.10) *0.037* *0.60* 0.67 (0.26) 1.71 (1.16) 1.41 (1.02) *0.094 0.81*

Legumes 0.75 (0.27) 0.85 (0.69) 1.07 (0.80) *0.42 0.95* 1.58 (1.11) 0.83 (0.25) 1.04 (0.81) *0.31 0.86* 0.67 (0.26) 1.04 (0.66) 0.77 (0.25) *0.17 0.81*

Plant milks 0.50 (0.00) 0.88 (0.94) 0.85 (0.86) *0.56 0.95* 0.92 (1.02) 0.50 (0.00) 0.63 (0.53) *0.49 0.86* 3.58 (7.55) 0.92 (0.97) 0.64 (0.54) *0.72 0.93*

Low-fat yogurt 1.83 (1.29) 2.31 (5.06) 2.28 (3.82) *0.58 0.95* 0.92 (1.02) 1.22 (1.03) 2.00 (3.83) *0.34 0.86* 1.75 (1.37) 2.83 (5.19) 1.98 (3.94) *0.52 0.93*

Low-fat milk 1.3 (1.3) 6.4 (8.8) 2.9 (5.2) *0.70 0.96* 1.8 (1.3) 1.9 (1.3) 5.3 (7.5) *0.84 0.95* 4.8 (7.0) 8.7 (9.2) 2.6 (3.8) *0.45 0.93*

Other fruits 8.3 (8.3) 15.3 (7.0) 12.7 (8.0) *0.20 0.95* 8.0 (8.6) 13.7 (8.0) 11.9 (8.3) *0.37 0.86* 11.0 (8.8) 12.1 (8.5) 10.1 (8.3) *0.81 0.93*

Other vegetables 2.2 (1.2) 6.7 (7.0) 11.3 (8.3) *0.013* *0.34* 2.7 (0.8) 7.9 (8.4) 7.7 (7.7) *0.38 0.86* 5.0 (6.9) 4.3 (4.6) 4.3 (4.8) *0.54 0.93*

Crucifers 1.08 (0.97) 1.35 (0.97) 1.09 (0.79) *0.53 0.95* 1.17 (0.93) 1.11 (0.74) 1.02 (0.82) *0.56 0.86* 0.83 (0.26) 1.33 (1.03) 1.00 (0.69) *0.75 0.93*

Quality score 9.2 (3.8) 10.2 (4.8) 10.5 (3.8) *0.84 0.98* 10.5 (7.1) 8.4 (5.9) 9.3 (4.6) *0.82 0.95* 12.7 (4.4) 10.6 (6.3) 8.5 (4.2) *0.20 0.81*
