# Supplementary material for: Parity modulates impact of BMI and gestational weight gain on gut microbiota in human pregnancy
Source: Gut Microbes. 2023 Oct 9;15(2):2259316. doi: 10.1080/19490976.2023.2259316 (PMC10563629; doi:10.1080/19490976.2023.2259316)
Supplement: Supplemental Material [file KGMI_A_2259316_SM6322.zip › Supplementary files/TableS3clean.docx]

**Supplemental Table 3. Impact of parity on diet**

First trimester

Second trimester

Third trimester

**Primiparous***^1^*

**Multiparous***^1^*

**p***2*

**q***3*

**Primiparous***^1^*

**Multiparous***^1^*

**p***2*

**q***3*

**Primiparous***^1^*

**Multiparous***^1^*

**p***2*

**q***3*

Weekly servings

n

30

13

23

15

25

16

Green vegetables

0.82 (0.25)

2.27 (5.03)

*0.26*

*0.75*

1.65 (3.79)

1.97 (4.72)

*0.29*

*0.81*

0.86 (0.23)

1.91 (4.57)

*0.48*

*0.66*

Carrots

1.52 (1.09)

5.00 (6.27)

*0.002*

*0.062*

1.67 (1.10)

2.43 (0.98)

*0.042*

*0.55*

1.90 (1.09)

3.31 (4.31)

*0.22*

*0.62*

Citrus

2.4 (4.6)

3.4 (4.8)

*0.031*

*0.27*

4.5 (6.9)

6.1 (8.1)

*0.79*

*0.90*

4.5 (6.5)

5.6 (8.0)

*0.47*

*0.66*

Whole dairy

9.9 (8.7)

8.8 (8.4)

*0.92*

*0.96*

12.4 (8.5)

12.5 (8.3)

*0.84*

*0.91*

7.0 (7.7)

10.6 (8.7)

*0.29*

*0.62*

Low-fat greek yogurt

0.60 (0.20)

0.54 (0.14)

*0.33*

*0.86*

0.57 (0.17)

0.80 (0.65)

*0.13*

*0.67*

0.56 (0.17)

1.91 (4.60)

*0.11*

*0.58*

Cottage cheese

0.67 (0.48)

2.15 (5.11)

*0.70*

*0.90*

0.78 (0.72)

0.90 (0.87)

*0.72*

*0.90*

0.90 (0.94)

1.03 (0.99)

*0.34*

*0.62*

Eggs

1.70 (1.10)

1.85 (1.13)

*0.68*

*0.90*

1.57 (0.98)

1.47 (0.97)

*0.71*

*0.90*

1.32 (0.88)

1.81 (1.09)

*0.18*

*0.62*

Nuts

3.6 (5.3)

2.5 (5.0)

*0.052*

*0.34*

3.5 (5.0)

2.8 (4.6)

*0.40*

*0.81*

3.4 (6.0)

4.1 (6.0)

*0.41*

*0.62*

Processed meat

1.05 (0.91)

1.69 (1.09)

*0.016*

*0.21*

1.37 (1.12)

1.60 (1.20)

*0.57*

*0.90*

1.48 (1.18)

1.94 (1.11)

*0.089*

*0.58*

Seafood

1.18 (0.95)

1.08 (0.61)

*0.39*

*0.90*

0.96 (0.69)

1.17 (0.77)

*0.17*

*0.68*

0.76 (0.25)

0.94 (0.60)

*0.41*

*0.62*

Whole grains

8.1 (7.8)

11.0 (9.0)

*0.69*

*0.90*

6.9 (7.4)

10.2 (8.5)

*0.32*

*0.81*

6.0 (6.7)

11.9 (8.4)

*0.029*

*0.58*

Sweetened drinks

2.05 (3.39)

2.81 (4.99)

*0.77*

*0.91*

2.8 (5.2)

2.2 (4.7)

*0.30*

*0.81*

2.24 (3.65)

1.34 (1.17)

*0.15*

*0.62*

Salt

3.8 (6.2)

0.8 (0.7)

*0.066*

*0.34*

2.72 (5.24)

1.53 (1.25)

*0.88*

*0.92*

1.96 (3.72)

1.03 (0.99)

*0.59*

*0.76*

*1* N; Mean (SD)

*2* Wilcoxon rank sum test

*3* False discovery rate correction for multiple testing

Fried foods 0.68 (0.25) 0.77 (0.70) *0.51 0.90* 0.72 (0.25) 0.77 (0.65) *0.40 0.81* 0.76 (0.52) 0.78 (0.63) *0.82 0.90*

Baked goods 2.60 (4.57) 1.42 (1.12) *0.53 0.90* 3.04 (3.62) 1.60 (1.04) *0.042* *0.55* 3.78 (4.67) 1.97 (1.07) *0.071 0.58*

Refined grains 2.8 (4.5) 3.2 (4.9) *0.74 0.91* 3.4 (5.0) 3.1 (4.5) *>0.99 >0.99* 2.7 (3.6) 4.6 (7.2) *0.40 0.62*

Poultry 1.38 (1.01) 1.46 (1.09) *0.91 0.96* 1.50 (1.14) 2.50 (4.65) *0.60 0.90* 1.64 (1.15) 1.53 (1.04) *0.92 0.92*

Red meat 1.28 (0.99) 1.85 (1.13) *0.094 0.37* 1.52 (1.02) 1.93 (1.19) *0.50 0.87* 1.30 (1.00) 1.63 (1.12) *0.34 0.62*

Legumes 0.97 (0.73) 0.92 (0.67) *0.98 0.98* 0.87 (0.53) 1.40 (1.02) *0.11 0.67* 0.78 (0.25) 0.91 (0.61) *0.83 0.90*

Plant milks 0.85 (0.86) 0.69 (0.69) *0.47 0.90* 0.61 (0.52) 0.70 (0.65) *0.35 0.81* 0.70 (0.69) 1.84 (4.62) *0.32 0.62*

Low-fat yogurt 2.52 (4.60) 1.62 (1.16) *0.64 0.90* 1.85 (3.85) 1.33 (1.06) *0.45 0.83* 2.74 (5.01) 1.28 (1.05) *0.92 0.92*

Low-fat milk 4.4 (6.7) 2.3 (5.1) *0.10 0.37* 3.2 (5.1) 5.0 (7.3) *0.76 0.90* 5.4 (7.0) 4.7 (7.2) *0.30 0.62*

Other fruits 12.6 (8.0) 14.1 (7.7) *0.58 0.90* 10.4 (8.5) 13.7 (7.8) *0.18 0.68* 9.1 (8.2) 14.0 (7.7) *0.053 0.58*

Other vegetables 8.2 (7.8) 9.0 (8.2) *0.81 0.91* 6.8 (7.4) 7.1 (7.4) *0.63 0.90* 4.1 (4.5) 5.8 (6.6) *0.68 0.81*

Crucifers 1.07 (0.81) 1.35 (0.97) *0.25 0.75* 0.98 (0.68) 1.20 (0.96) *0.74 0.90* 1.14 (0.86) 0.94 (0.60) *0.67 0.81*

Quality score 10.0 (4.1) 10.8 (4.0) *0.57 0.90* 8.2 (5.4) 11.0 (4.7) *0.11 0.67* 8.8 (5.0) 11.1 (4.8) *0.27 0.62*
