# Supplementary figures and images for: Parity modulates impact of BMI and gestational weight gain on gut microbiota in human pregnancy
Source: Gut Microbes. 2023 Oct 9;15(2):2259316. doi: 10.1080/19490976.2023.2259316 (PMC10563629; doi:10.1080/19490976.2023.2259316)

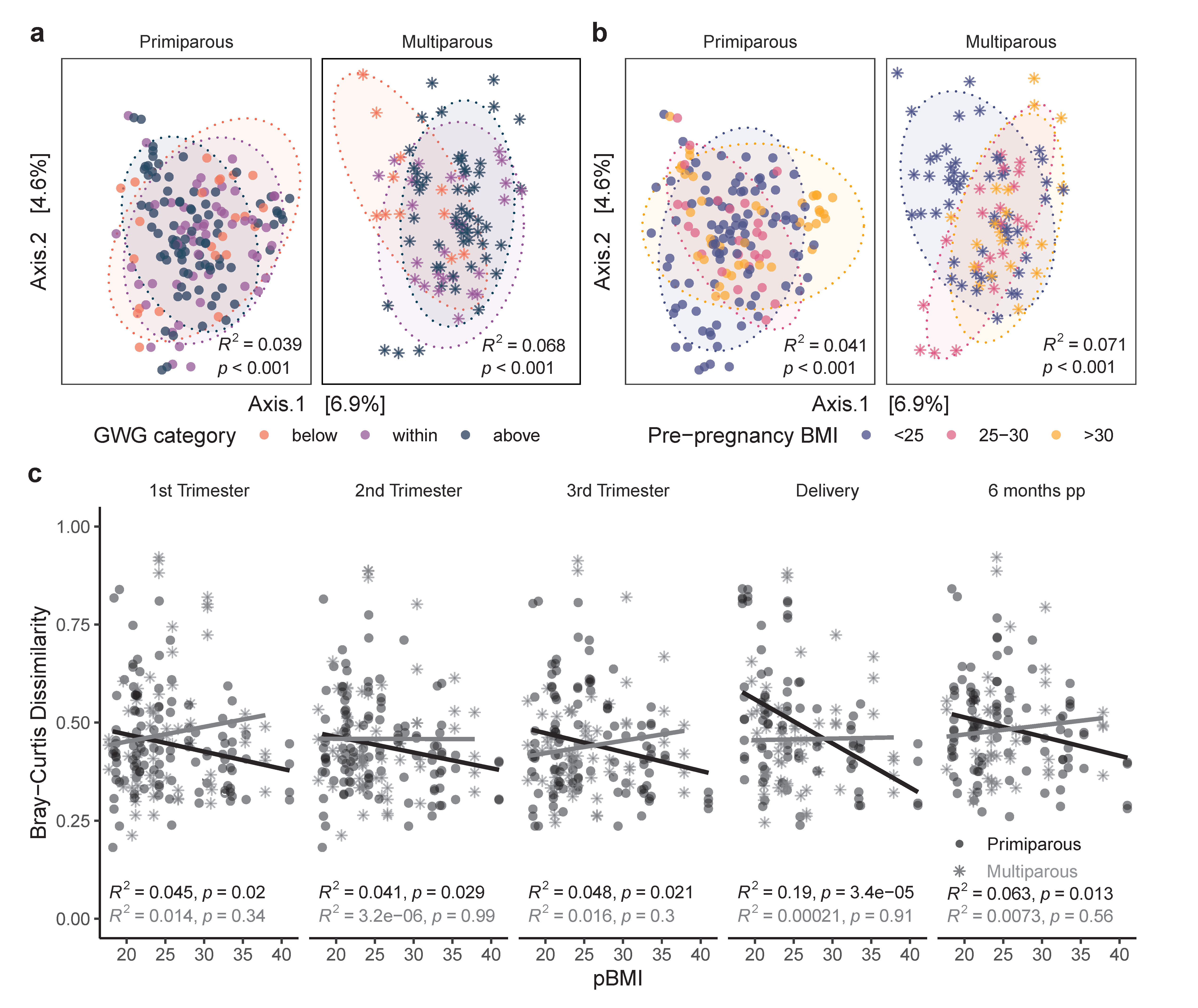

Supplement: Supplemental Material [file KGMI_A_2259316_SM6322.zip › Supplementary files/SupplementalFig1.tif]

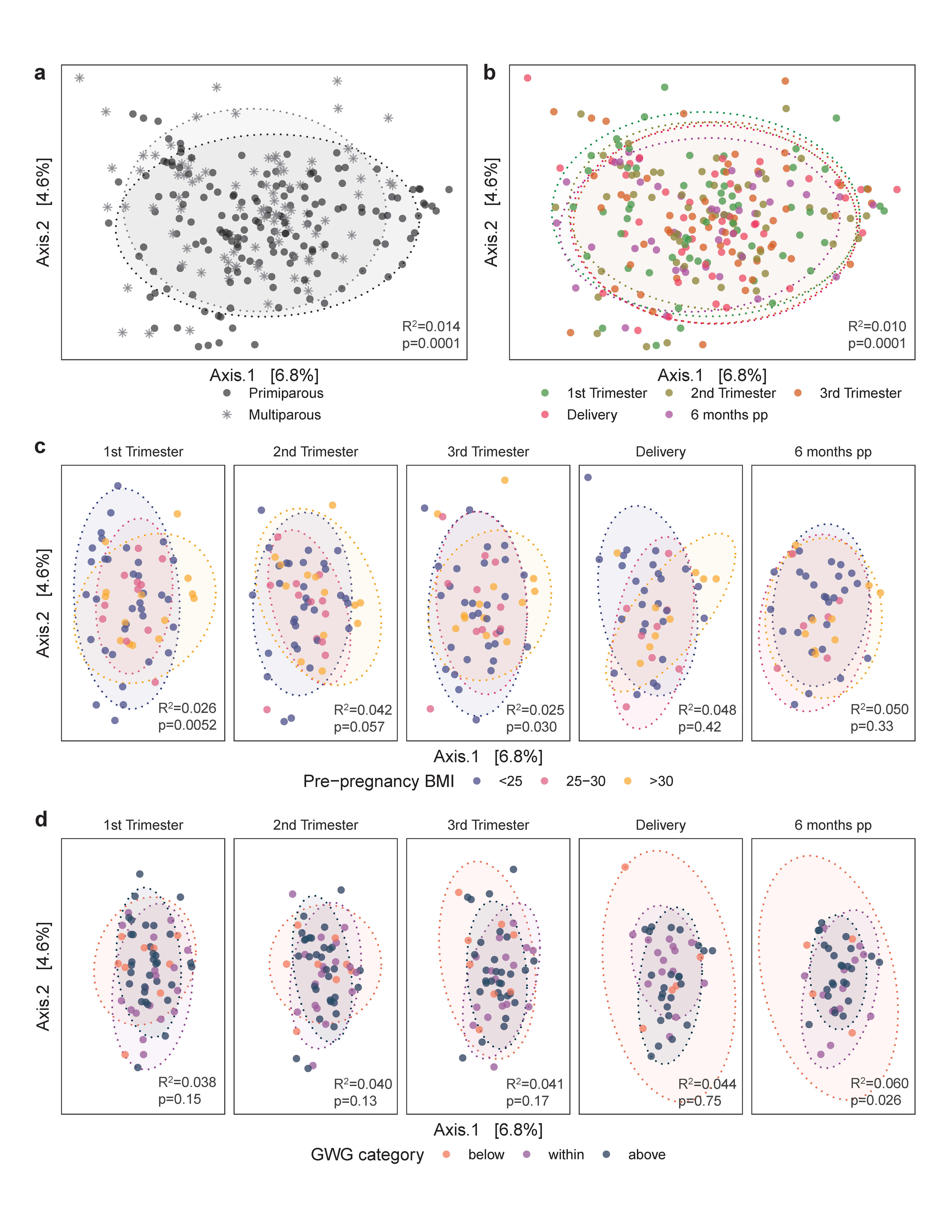

Supplement: Supplemental Material [file KGMI_A_2259316_SM6322.zip › Supplementary files/SupplementalFig2.tif]

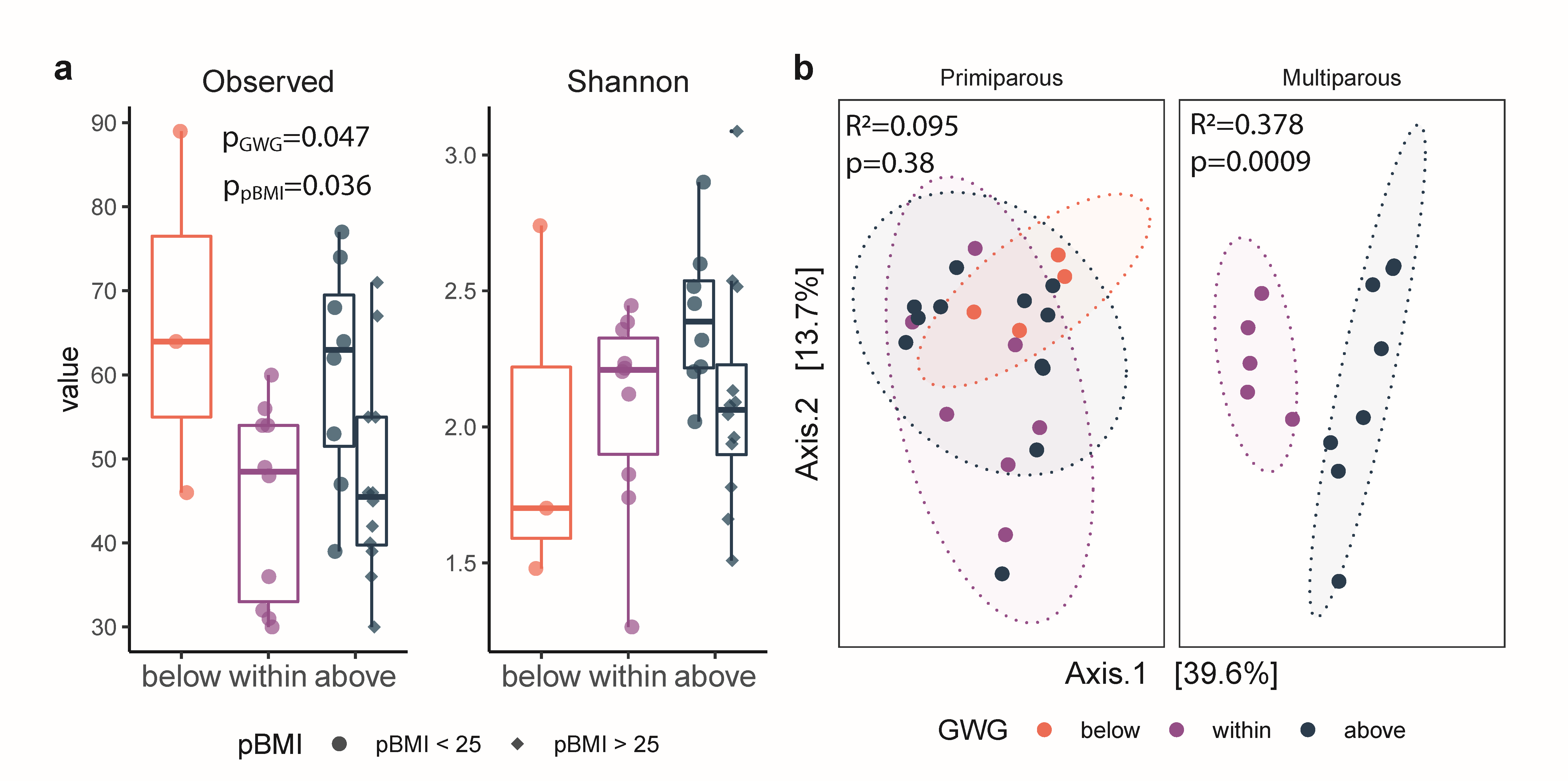

Supplement: Supplemental Material [file KGMI_A_2259316_SM6322.zip › Supplementary files/SupplementalFig5.tif]
